# Looking at Buswell’s pictures

**DOI:** 10.16910/jemr.13.2.4

**Published:** 2020-06-02

**Authors:** Nicholas J. Wade

**Affiliations:** Psychology, University of Dundee, Dundee DD1 4HN, UK

**Keywords:** eye movements, art perception, attention, individual differences, Buswell, Stratton, Judd, Yarbus

## Abstract

In 1935 Guy Buswell, an educational psychologist at Chicago University, published How People Look at Pictures. In it he recorded photographically the eye movements of 200 observers when looking at a wide variety of pictures. He analysed the overall distribution of fixations on pictures, compared the first few fixations on a picture to the last few, measured the durations of fixations made early in viewing and those made near the end of viewing, examined how fixation duration changed with viewing time, recorded the consistency between different observers when viewing the same picture and he looked at the influence of instructions given to observers upon their eye movements when viewing a picture. He commented on the substantial differences between individuals and noted that instructions had a dramatic effect on the pattern of eye movements. Buswell’s analysis was graphical rather than statistical. In this article Buswell’s figures are recombined and his research is placed in the context of earlier investigations of eye movements with pictures by Stratton and Judd and later ones by Yarbus.

## Introduction

“The effect of different types of design in carrying the eye swiftly from one place to another is apparently much less than is assumed in the literature of art.” (Buswell, 1935, p. 115, 5)

**Figure 1.**
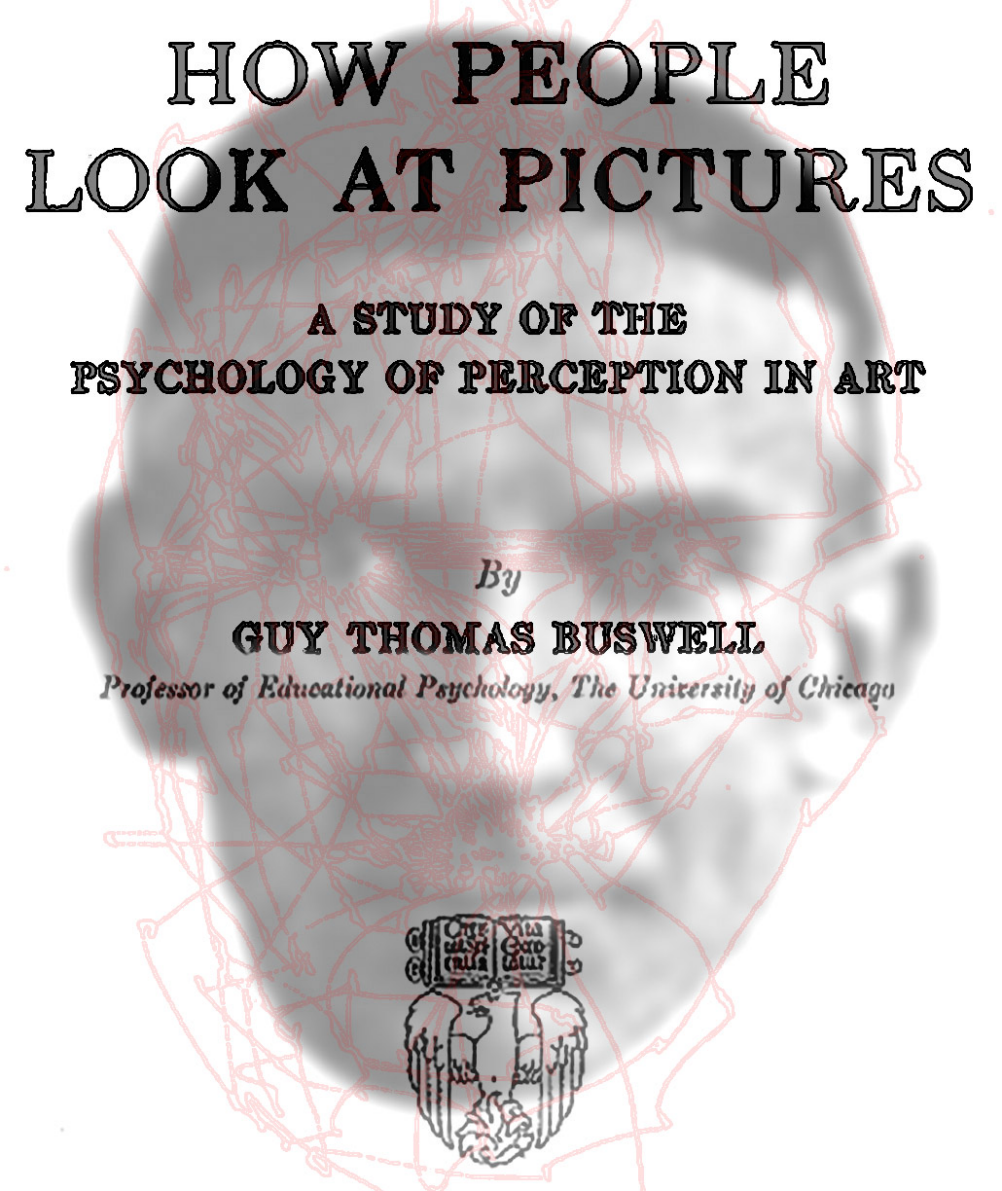
*Buswell’s book*. (Portrait adapted from an illustration in Taylor, 23, and a conventional photographic copy can be found in Wade & Tatler, 30; eye movement trace adapted from Yarbus, 32.)

In 1935 Guy Thomas Buswell (Figure 1) published his investigations on an issue that had exercised the minds of commentators on art for many years – how the eyes move when observing pictures (see 18). Indeed, he wrote: “Many books on the subject of art make reference to eye movements. The statements which are made rest on introspective evidence, but they indicate an acute interest on the part of the artist and the art critic in the nature of the process of visual perception” (5, p. 7). He then gave ten examples drawn from books earlier in the twentieth century after which he added: “The data furnished in the following pages will make it possible to observe the extent to which the various hypotheses regarding eye movements are in harmony with the objective patterns of perception that will be shown” (p. 9). Buswell’s book is well illustrated and many of his arguments are made graphically rather than statistically. His approach and conclusions are described and displayed in this article. The pictures Buswell presented are printed in an appendix; the locations of eye fixations, their sequences, paths and distributions over selected pictures are printed in the text. Some of these are re-presented in colour because the grey scale originals are of low contrast and are difficult to reproduce. This enables several of the original density maps and eye tracks to be incorporated into single illustrations. Buswell’s pioneering study is placed in the context of earlier investigations by Stratton and Judd as well as the later studies of Yarbus.

Guy Buswell was born in Lincoln, Nebraska in 1891 and died there 103 years later. As an undergraduate at York College, Nebraska, he was influenced by Hugo Munsterberg’s book on applied psychology ( 16) and continued his education in psychology at the University of Chicago. Many of the eminent early American psychologists were active in the department and the one who directed Buswell’s research most was Charles Hubbard Judd (1873-1946). Judd had studied under Wundt and subsequently pursued investigations of the movement sense in the context of visual illusions. These were examined with the aid of a movie camera system for registering eye movements. Buswell completed a PhD under the supervision of Judd. Buswell’s initial research interest was in reading along with his work on learning and teaching arithmetic, for which he is particularly famous (2, 3, 4, 6, 11). The apparatus at Chicago was under constant development (23) and Buswell adapted it to examine eye movements in reading. The eye movement system used for his studies of pictures was designed and built for him in the education department of the University of Chicago. He remained in the department until 1949 when he was appointed to the University of California, Berkeley where he remained until he retired in 1958 (see 10). Buswell was highly regarded by his peers and his students. He was initiated into the Reading Hall of Fame in 1974 and has been considered as one of the pioneers of eye movement research (26; 30).

### Buswell’s book

**Figure 2.**
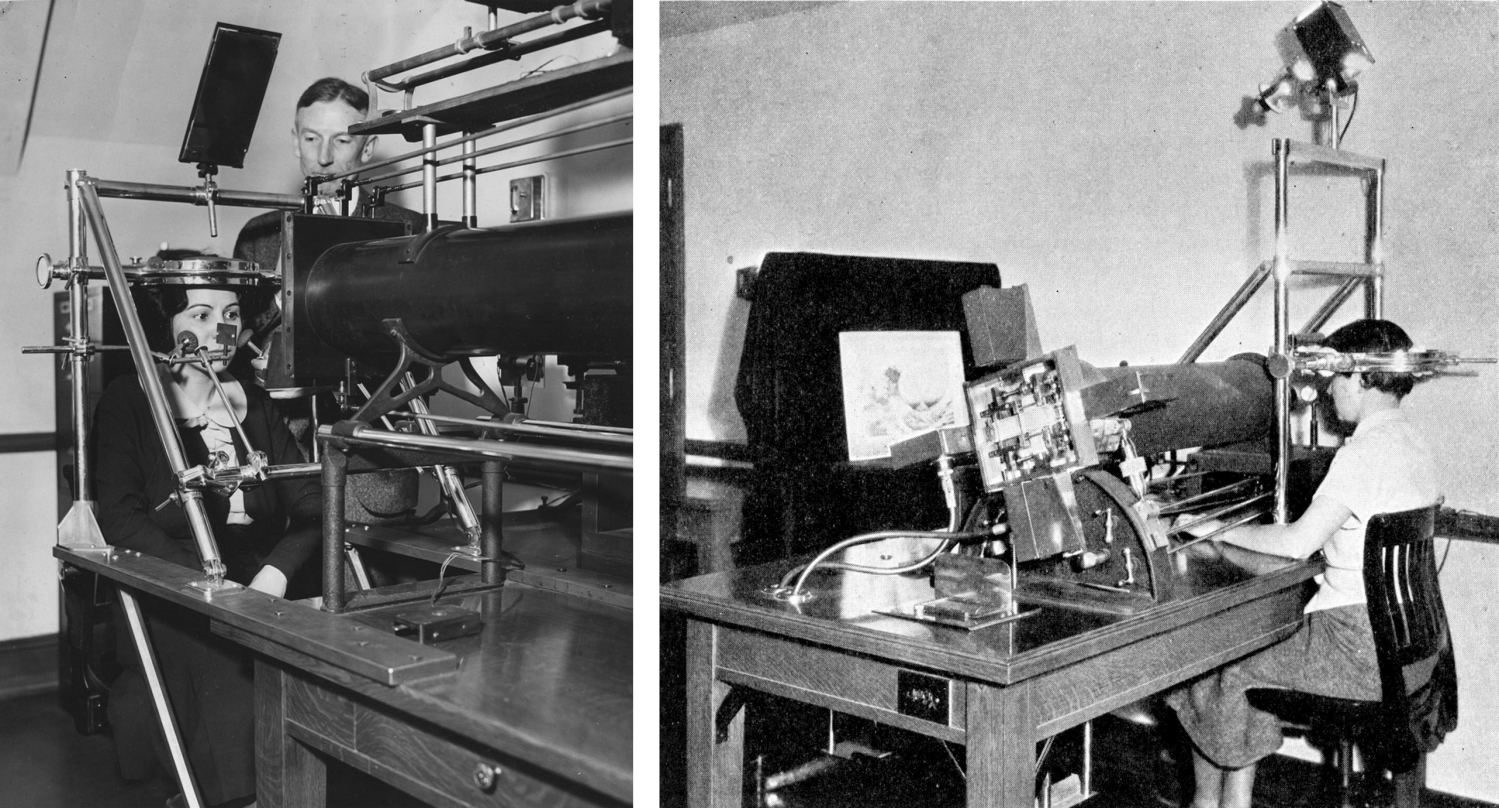
Left, Buswell looking over a participant in a study of eye movements; the photograph is undated and it probably shows apparatus for recording eye movements during reading. (Photograph courtesy of University of Chicago Photographic Archive, [apf1-02381], Special Collections Research Center, University of Chicago Library.) Right, Buswell’s apparatus specially constructed for photographing eye movements when viewing pictures; the picture presented to the observer is *The Wave* by Hokusai. (Plate V in Buswell, 5.)

In his book Buswell acknowledged that eye movements in reading had been investigated intensively using photographic methods but his technique was novel in its application to art. The technical difficulties imposed by this task were recording vertical as well as horizontal eye movements and the apparatus he constructed for this purpose was somewhat cumbersome (Figure 2). He used a corneal reflection eye tracker to study eye movements of observers as they viewed pictures and geometric patterns. The camera recording the light reflected from the surface of the cornea was positioned at right angles to the

subject so that they had a clear field of view for observing images placed in front of them. Head movements were also recorded using this device; this was to correct the eye position record for any slight movements of the subject’s head during viewing. Head movements were measured by recording the reflection of light from a chromium bead placed on a pair of glasses worn by the observer. In his experiments using this eye tracking device, Buswell found that eye movements cluster around regions of interest in pictures and that there was greater consistency between observers early in viewing than after several minutes of viewing the same picture.

Buswell reports eye movement data recorded from 200 participants each viewing multiple pictures, such that his data comprised almost two thousand eye movement records each containing a vast number of fixations. The participants were drawn from a wide population: 12 elementary and 44 high school pupils, and 144 adults. The majority of the adults were college and graduate students and 47 were recruited from the Art School of the Art Institute of Chicago. Not all participants viewed all pictures with a range from 12 to 68. Most of the 55 illustrations he presented to participants were photographs of items in the collection of the Art Institute of Chicago; all are printed as numbered Pictures in an Appendix. Buswell categorised the prints and photographs as: art works (16), vases and dishes (6), furniture and design (8), statues (5), tapestries (4), architecture (8), posters (3) and geometrical figures (5). Eye movement records are printed as Plates in the text and accumulated data in tables.

The volume of eye movement data collected by Buswell is impressive by modern standards, but particularly so given the technology at the time and the need to transform manually the horizontal and vertical position of the eye indicated in the eye movement records into precise locations on the pictures viewed. This work was the first to explore systematically eye movements while viewing complex pictures, rather than text or simple patterns. Using complex images and scenes has become a central aspect of modern eye movement research and is an important part of understanding eye movements in everyday vision (1; 13; 14).

Buswell explored a wide range of issues regarding the eye movements made while viewing pictures, including some surprisingly modern concerns: he looked at the overall distribution of fixations on pictures; he compared the first few fixations on a picture to the last few; he compared the durations of fixations made early in viewing to those made near the end of viewing; he looked at how fixation duration changed with viewing time; he compared the consistency between different observers when viewing the same picture; and he looked at the influence of instructions given to observers upon their eye movements when viewing a picture.

Five major aspects of the eye movement records were considered in sequence by Buswell: centres of interest, fixation durations, picture characteristics, individual differences and the effects of instructions. These will be described and illustrated where possible with respect to the picture illustrated most frequently in Plates by Buswell – Hokusai’s *The Wave*.

Density plots were made of where all participants fixated when viewing pictures and showed that not all locations and objects in pictures are fixated, with particular “centers of interest” where fixations are concentrated. He also appreciated that there could be quite large individual differences in where people fixate when viewing pictures:

“The positions of the fixations indicate clearly that for certain pictures the center or centers of attention are much more limited than in other pictures. The fact that the density plots represent composite diagrams from a great many different subjects obviously results in a wider distribution of fixations on account of the varied interests of different individuals in looking at the same picture. However, the density plots do give a rather clear indication as to what parts of a given picture are likely to prove most interesting to a random selection of subjects.” (5)

**Figure 3.**
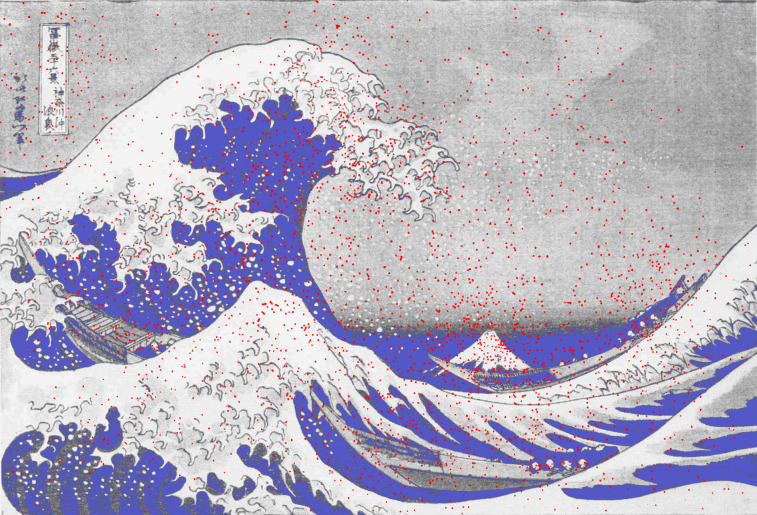
*Waves of eye movements 1*. Density plots for all fixations of 42 observers when viewing *The Wave* by Hokusai. Fixations are denoted by red dots. (Adapted from Picture 13 and Plate XI from Buswell, 5.)

The density plots for eye movements over *The Wave* are shown in Figure 3. Buswell noted that “The large wave and the small white mountain are probably the main centers of interest” (p. 20).

**Figure 4.**
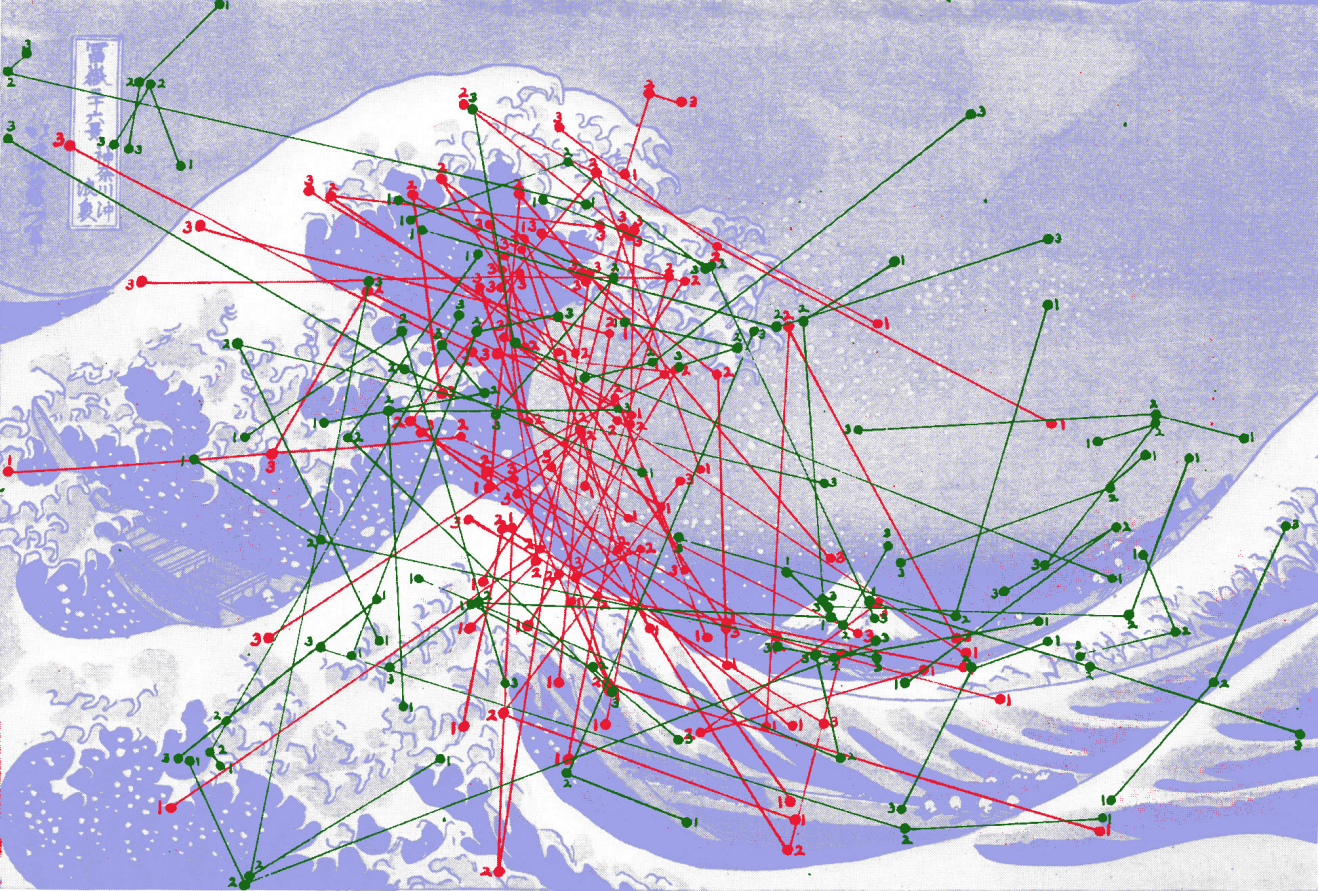
*Waves of eye movements 2*. Eye movement records when viewing *The Wave* by Hokusai. Each circle represents a fixation made by an observer. The lines indicate the saccades that moved the eye from one fixation to the next. The first three fixations (numbered in sequence) from the start of viewing for 40 observers are shown in red and the final three fixations in green. (Adapted from Picture 13 and Plates XIV and XV in Buswell, 5.)

Buswell acknowledged that the wide distribution of fixations is a consequence of the number of observers and so carried out a more detailed analysis of individual patterns of eye movements early and late in a trial; there was a greater concentration of fixations initially compared to the later diversity and detailed inspection (Figure 4).

**Figure 5.**
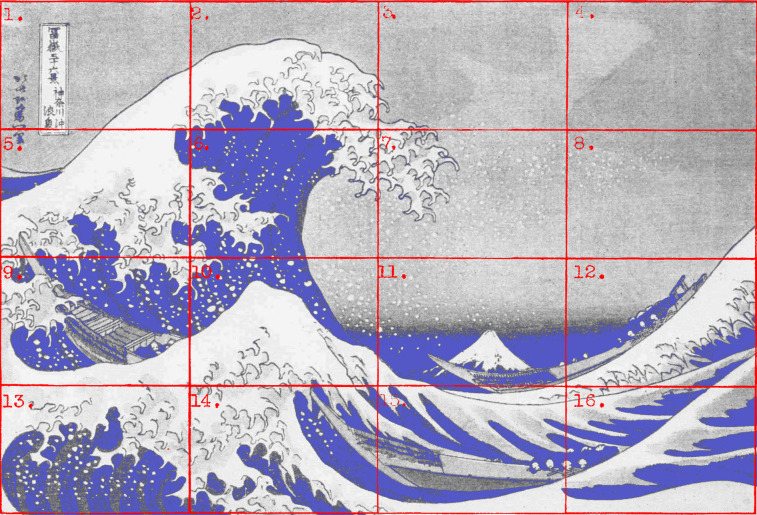
A grid of 16 rectangles superimposed on Hokusai’s *The Wave*. (Adapted from Picture 13 and Plate XVI in Buswell, 5.)

Buswell sought to make more detailed comparisons by sectioning the pictures into 16 equal rectangles (Figure 5) and examining the percentage of fixations falling in each one.

**Figure 6.**
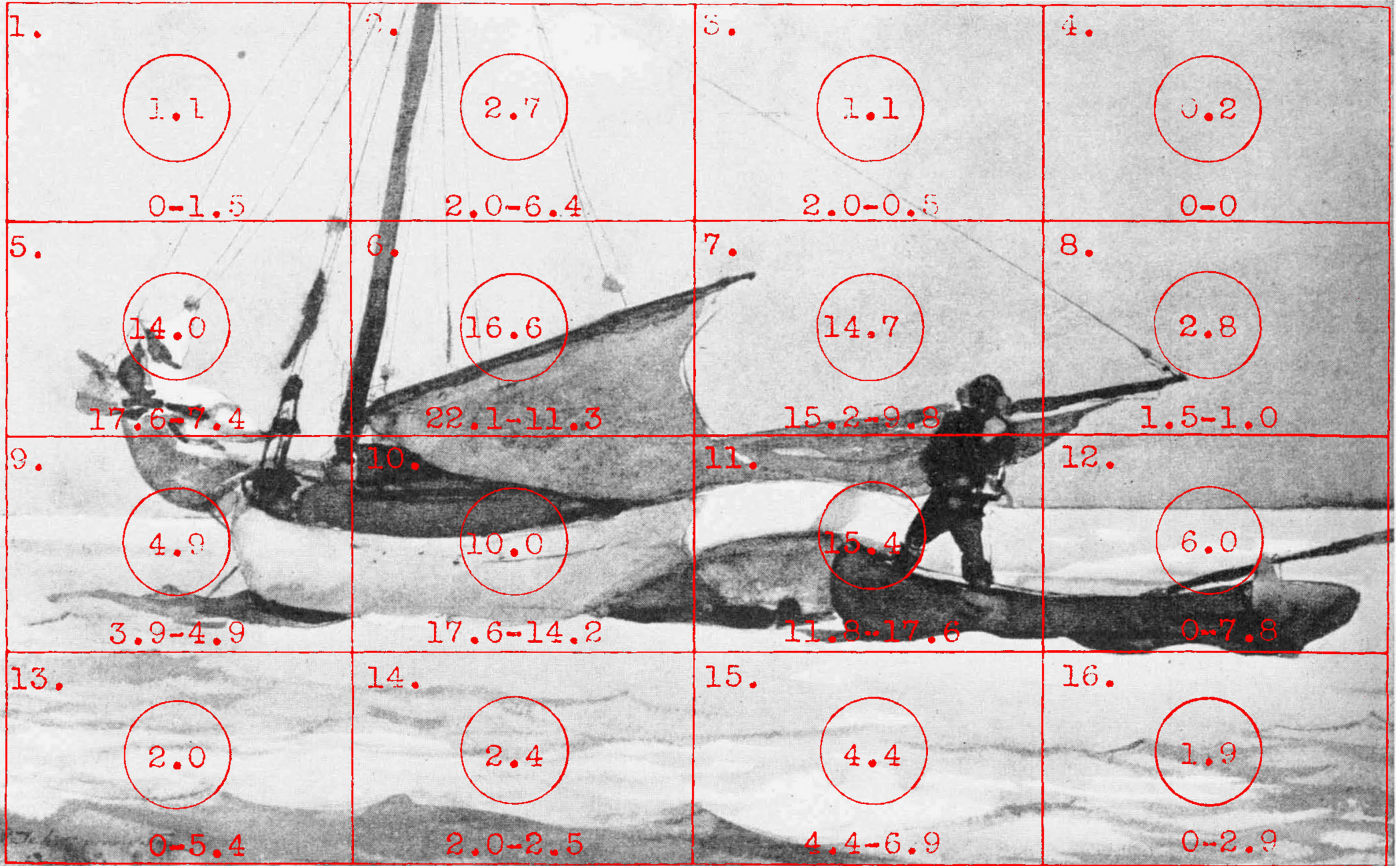
*Regional variations*. A grid of 16 rectangles superimposed on a photograph of *Stowing the sail – Bahamas* by Winslow Homer. Numbers in the circles represent the percentage of the first 18 fixations which fall in each rectangle; numbers separated by dash indicate the percentage of the first three and last three fixations falling in each rectangle. (Adapted from Picture 9 and Plate XVI in Buswell, 5.)

There are obvious limitations in this procedure because of the large structural differences between the pictures. An example is shown in Figure 6 for a photograph of a painting.

**Figure 7.**
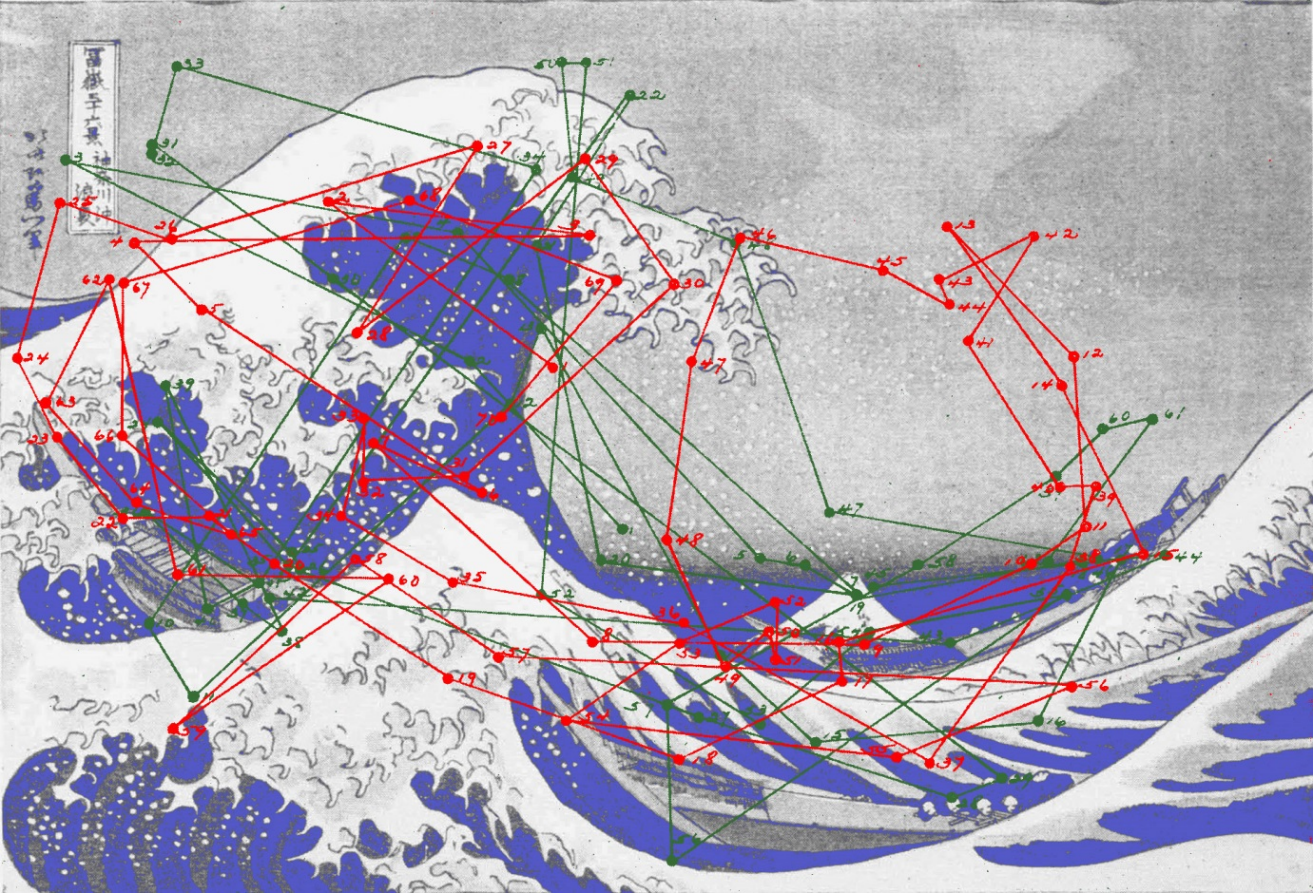
*Waves of eye movements 3*. Eye movement records when viewing *The Wave* by Hokusai. Each colour (red and green) represents the sequential fixations made by two observers. The lines indicate the saccades that moved the eye from one fixation to the next. (Adapted from Picture 13 and Plates XXXII and XXXIII in Buswell, 5.)

In addition to the general features of eye movements over the pictures, Buswell selected certain individual records so that the generality of the grouped data did not mask idiosyncratic characteristics. Unfortunately, Buswell does not provide the criteria for the selections he makes. It is of interest to note that fixation is explicitly equated with perception: “a series of individual records will be shown in which the actual patterns of perception are presented with objectivity and precision” (p. 45). Two such were presented for viewing *The Wave* (Figure 7). In describing the sequence shown in red, in which the eye tracking follows the sweep of the large wave, Buswell writes “The manner in which this particular subject looked at the picture furnishes an excellent example of how an artist is able to control the perceptual process by the composition of his picture” (p. 59). The other record in green is said to indicate how the observer concentrates on detail.

**Figure 8.**
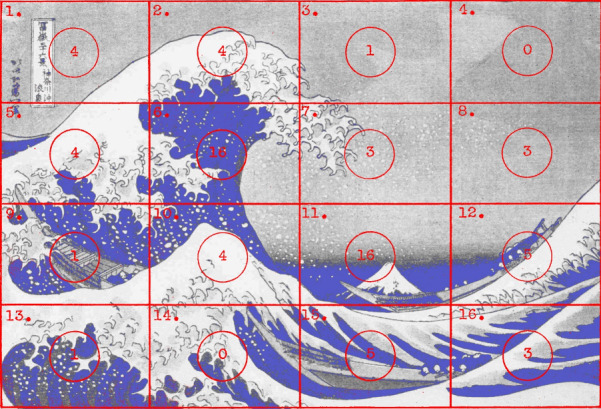
*Waves of eye movements 4*. Eye movement records when viewing *The Wave* by Hokusai. The distribution of the number of long fixations (greater than 667 ms) for 38 observers over the picture. (Adapted from Picture 13 and Plate XLVII in Buswell, 5.)

The durations of fixations varied considerably with the average over all pictures for 29 observers being around 300 ms. They differed between individuals and pictures and within individuals (increasing during a trial) and pictures (dependent on content). The distribution of

long fixations over *The Wave* is shown in Figure 8. The regions with the most long fixations (the large wave and mountain) were also those that had the largest proportion of all fixations (see Figure 3).

With regard to picture characteristics Buswell found that colour had little effect on the position or durations of fixation. The pattern of eye movements over repetitive designs “does not resemble even remotely the general pattern of the design” (p. 143).

The issue of individual differences in the patterns of eye movements was explored in detail in a section at the end of the second chapter. The differences that were present in the locations fixated by individuals when viewing each image were also reflected in the durations of the fixations, with a large variation between observers in their average fixation duration on each picture. Buswell’s investigation of individual differences extended to exploring differences between artistically trained individuals and those without training; between children and adults; and between Western and Oriental participants. In all cases, differences between the groups were small: “The average differences between the groups were so much less than the individual differences within each group that the results cannot be considered significant” (5). Differences were found in the eye movement data that emerged over the course of viewing a picture for some time. The regions fixated in the picture were more consistent between observers for the first few fixations than for the last few on each picture. Buswell also found that fixation duration increased over the course of viewing a picture for some time.

Buswell devoted a chapter of the book to looking at the influence of the characteristics of the picture upon location of fixation. This work is very reminiscent of that conducted some years earlier by George Malcolm Stratton (1865-1957) although he did not cite Stratton’s work. In places Buswell appears to argue that eye movements do tend to follow lines in pictures. This is contrary to Stratton’s suggestion that eye movements do not appear to be particularly influenced by the form of the figure being viewed. However, other aspects of Buswell’s data suggest less concordance between eye movements and the characteristics of the picture. When he showed participants more basic designs and patterns he found that: “The effect of different types of design in carrying the eye swiftly from one place to another is apparently much less than is assumed in the literature of art. … The writer should emphasize that the data from eye movements are not to be considered as evidence either positively or negatively for any type of artistic interpretation” ( 5). Like Stratton, Buswell felt that the pattern of eye movements was insufficient to explain our visual experience and so highlighted the need to appeal to cognitive explanations of vision.

**Figure 9.**
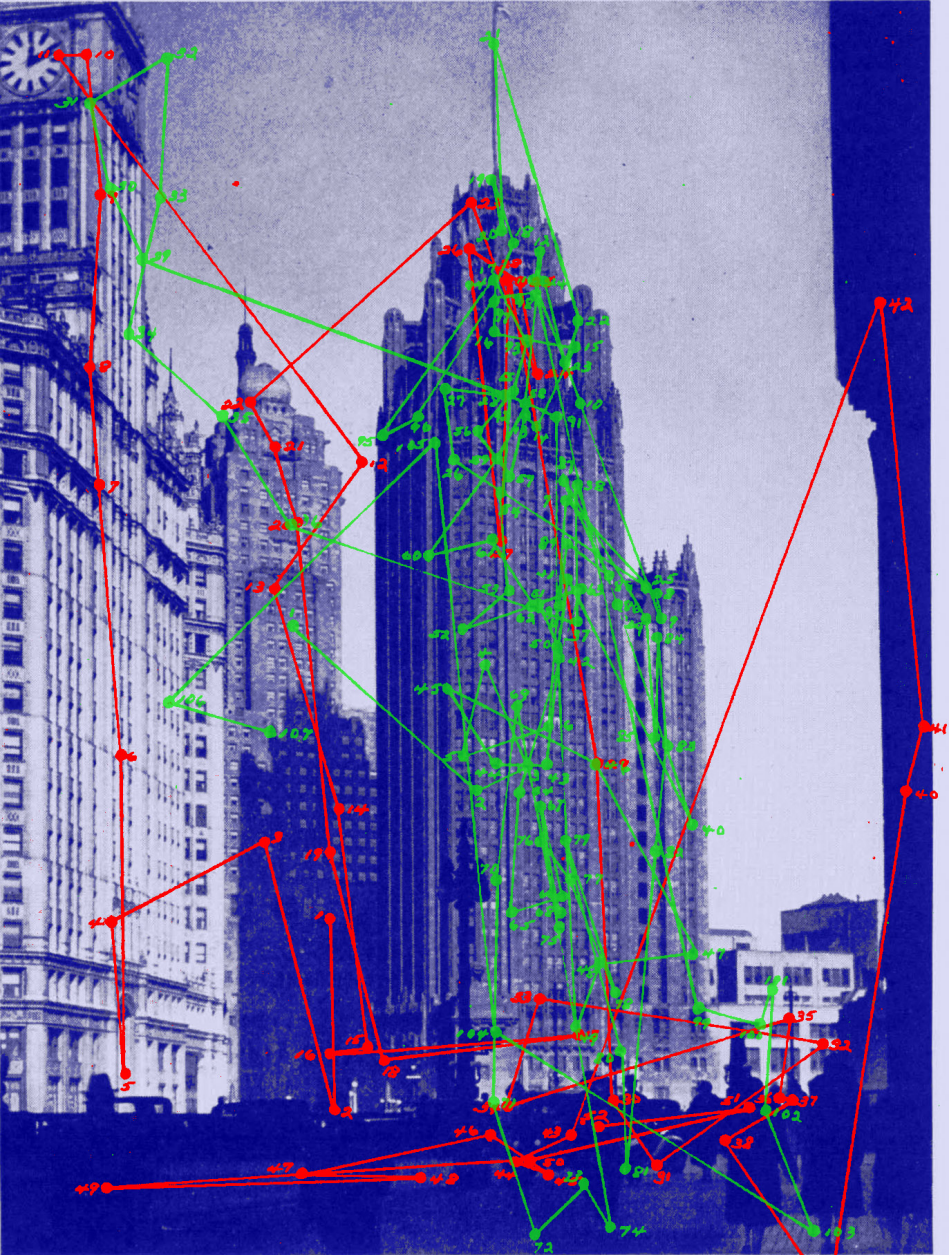
*Directed viewing*. Eye movements of one observer viewing a picture of the Chicago Tribune Tower without specific instructions (red) followed by directions to find a person looking out of a window in the Tower (green). (Adapted from Picture 43 and Plates LXV and LXVI in Buswell, 5.)

The directions given to observers prior to looking at a pattern did have a marked influence on the characteristics of their eye movements. The example Buswell gave is of one observer asked to look at a picture of the Chicago Tribune Tower (Figure 9) “without any special directions being given. After that record was secured, the subject was told to look at the picture again to see if he could find a person looking out of one of the windows of the tower” (5). Both the locations of fixations as well as their durations were influenced by the instructions. The different eye movement records obtained in these two situations demonstrate that cognitive factors such as the viewer’s task can have a strong effect upon how a picture is inspected. Such descriptions of influences played by high level factors upon eye movements are not typically associated with Buswell, but rather it is Alfred Yarbus (1914-1986) who is generally regarded as the first to have offered such an observation (32).

A second method was applied to vary the directions for looking at pictures; after viewing a picture without any special instructions observers read descriptions of the pictures before viewing the picture again: “The page of material was designed to arouse interest in particular parts of the picture” (p. 139) Another group read the descriptions before looking at the picture. The results for this procedure were inconclusive in terms of the number of fixations, their durations and the centres of interest, although the pictures were observed for longer after reading.

## Earlier and later eye movement studies with pictures

### Stratton and Judd

**Figure 10.**
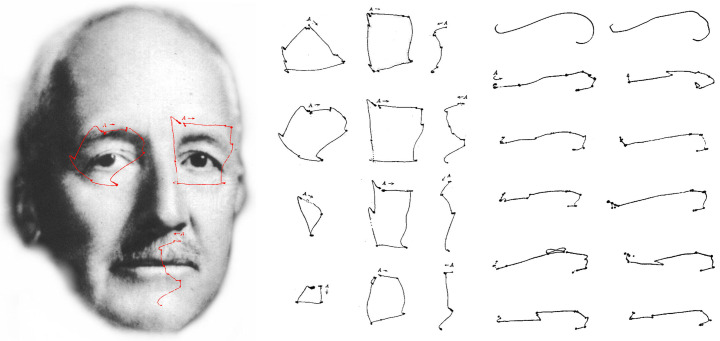
*Stratton’s symmetries*. Left, Stratton and his eye movement records (in red) for tracking a circle, a rectangle and an S-shape. Centre, the tracings of two observers when following the shapes (upper two) and those of the same observers when imagining the shapes (lower two). Right, an observer viewed the top two patterns on separate trials with the eye movements tracings shown below. (Portrait derived from a photograph kindly provided by Karen De Valois, and a conventional photographic copy can be found in Wade & Tatler, 30; eye movement patterns from Stratton, 20.)

Buswell’s starting point was the same as that adopted three decades earlier by Stratton (20; Figure 10) – descriptions by writers on art of the gracefulness of eye movements over curved shapes. Stratton, like Buswell after him, employed a photographic technique to examine eye movements when viewing patterns in order to examine the contention empirically. This was an important new direction for eye movement research and served to highlight the importance of saccades outside the context of reading. Like his contemporaries, Stratton was surprised by the discontinuity of eye movements: “The eye darts from point to point, interrupting its rapid motion by instants of rest. And the path by which the eye passes from one to another of these resting places does not seem to depend very nicely upon the exact form of the line observed” ( 20, p. 343). This quotation highlights a central aspect of Stratton’s work: he demonstrated that the path taken by the eye during saccades did not appear to relate to the form of the pattern viewed. Simple geometrical outline patterns were presented to observers. Stratton did not provide illustrations of them all but described them clearly in the text. The first studies used a circle, a rectangle with its proportions in the golden ratio and an S-shape made from two segments of circles. The eye movements of two observers when asked to trace the outlines are shown in the upper centre of Figure 10. The starting points for the traces are given by the letter A. In order to determine the distraction induced by the illustrations themselves, Stratton recorded eye movements when the same two observers viewed a black sheet of paper lying over the illustrations; their eye movements are shown in the lower central tracings of Figure 10. His conclusion was “The general course of the ocular movement over a graceful line is itself usually far from graceful” (1902, p. 345). How would this compare with the movements over a graceless curve? In order to investigate this Stratton compared eye movements over a smooth and an irregular curve. These are shown on the upper right of Figure 10, with the multiple tracings of eye movements of one observer displayed beneath the patterns: “It is at once seen that the records for the graceful line are not identical with those for the ungainly one. But we may certainly say that the contrasting groups of records are immeasurably more alike than are the two original curves with regard to their *aesthetic* character” (1902, p. 346). A similar pattern of eye movements was found for a second observer. When one of the observers viewed the tracings afterwards she was surprised by their irregularity which contrasted with the subjective impression of smooth movement.

Stratton (21) returned to this issue of the relationship between patterns and eye movements in a later article, which opened by saying that: “our pleasure in graceful curves could not be due to the ease and smoothness of the eye’s own motion in viewing these curves. For the ocular movement itself, when photographically recorded, is found to be interrupted and jerky and most unlike the figures we enjoy” (21, p. 82). The article went on to consider whether eye movements can explain simple visual illusions; and whether eye movements can explain the aesthetics of symmetry. Stratton found no evidence for eye movement explanations of the Müller-Lyer, Poggendorff, or Zöllner illusions.

In his investigation of the eye movements of observers while looking at a variety of symmetrical and non-symmetrical figures, Stratton was again surprised by what he found when he examined the photographic negatives recorded: “one is struck by the almost grotesque unlikeness between the outline observed and the action of the eye in observing it. For the most part the eye moves irregularly over the figure, seeking certain points of vantage from which the best view of important features may be obtained. And these positions are marked by the eye’s momentary resting there” (1906, p. 94). Again, the path taken by the eye during movements did not relate to the form of the figure being viewed, but Stratton also observed that the positions fixated did not show any clear and consistent relationship with the figure:

Now these points of rest are evidently of more consequence to the observer than the path by which the eye reaches them; indeed the form of any single path between two stops usually bears no observable resemblance to the outline which the subject was taking in, and which in many cases he believes his eye to be accurately following. But even these points of rest are not so arranged as to supply of themselves a rough sense of the form perceived, after the manner of an outline pricked disconnectedly in paper. The points of the eye’s rest in the records are usually too few and too inexact to give any such clear and connected perception of the form as the observer regularly and readily obtains. (21, p. 94)

Stratton seems to express some degree of despair concerning the lack of correspondence between the eye movements and the figure being observed. The degree of surprise and disbelief evident in this article highlights the fact that the saccade and fixate behaviour of the eye was still very much a new aspect of vision research. Not only was there no clear relationship between the eye movements and form of the figure viewed, but there was also no clear relationship between the symmetry of the figure viewed and the symmetry of the eye movements made when viewing it. Indeed, Stratton’s conclusion is “Introspectively it seems as if the eye’s movements were smooth and continuous, while the records show convincingly that its course is wild and broken. The illusion, I believe, arises from our confusing the point of attention with the point of ocular fixation” (1902, p. 349). This is contrary Buswell’s statement that fixation is equated with attention: “The underlying assumption in this study is that in a visual experience the center of fixation of the eyes is the center of attention at a given time” (1935, pp. 9-10).

Stratton’s work is significant because it attempts to bridge the gap between visual phenomena (symmetry and illusions), cognition (aesthetic judgements), and the underlying mechanisms (eye movements). This defined new directions for eye movement research, highlighting the discrepancy between cognition and perception in vision. Stratton explicitly stated the inadequacy of eye movements as an explanation for aesthetic judgements: “The sources of our enjoyment of symmetry, therefore, are not to be discovered in the form of the eye’s behaviour. A figure which has for us a satisfying balance may be brought to the mind by most unbalanced ocular motions” (1906, p. 95). It is clear from Stratton’s closing remarks in his article on eye movements and symmetry that he appreciated the gulf between perception and cognition and that he was aware that his work was defining new questions in vision that would be addressed in the future.

**Figure 11.**
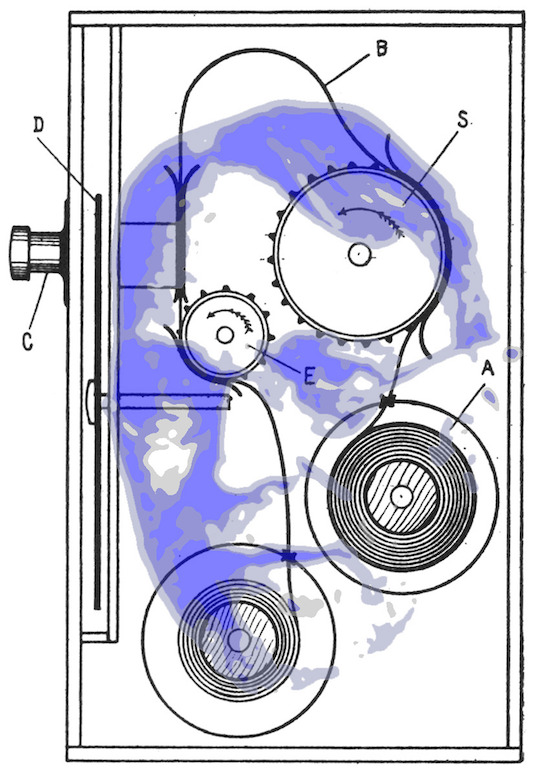
*Judd’s kinetoscope*. Judd and his movie camera system for recording eye movements. (Portrait adapted from a photograph in Murchison, 17, and a conventional photographic copy can be found in Wade & Tatler, 30; kinetoscope from Judd et al, 12.)

As stated earlier, Judd (Figure 11) had a great influence on Buswell at the University of Chicago, and introduced him to eye movement recording using a movie camera. Judd worked initially on geometrical illusions and was dismissive of interpretations based on angle expansion and perspective. He was impressed by the work of Dodge (9) and Stratton, but felt that the eye trackers used by both were somewhat limited in the range of tasks to which they could be applied: Dodge’s eye tracker was designed for recording movements only along a straight line (as occurs in reading) and Stratton’s lacked temporal resolution and required a dark room in which the photographic plate was exposed during experimental recording. Judd developed a ‘kinetoscopic’ eye tracker in which a small fleck of Chinese white (which had been affixed to the cornea) was photographed; eye movements could thus be discerned in two dimensions, and did not require a dark room in which to conduct the experiments (12). While Judd’s eye tracker offered specific advantages over previous photographic devices, it was still somewhat limited in its temporal resolution, typically operating at about 8-9 frames per second. One impressive feature of Judd’s eye tracker was that it allowed binocular recordings to be taken.

Judd’s interest in interpreting the results of the studies on illusions was in addressing the relationship between movement and perception (12). Judd sought to use his results to dismiss the notion that perceptions might arise directly from movements themselves; rather he stressed the importance of visual information from the retina both in forming perceptions and in coordinating the movements: “When the eye moves toward a point and the movement does not at first suffice to bring the point in question on the fovea, the retinal sensations which record the failure to reach the desired goal will be a much more powerful stimulus to new action than will any possible muscle sensation” ( 12, p. 218). While much of Judd’s discussion of his eye movement studies focused upon the discussion of their relation to theories of movement sensation, he did also notice that the pattern of eye movements was likely to be influenced by the instructions given to the observers during the experiments. The recognition that eye movements were not entirely directed by the stimulus being viewed echoed the opinion expressed by Stratton at the same time, but it was not investigated in any great depth by either Judd or Stratton.

### Yarbus

**Figure 12.**
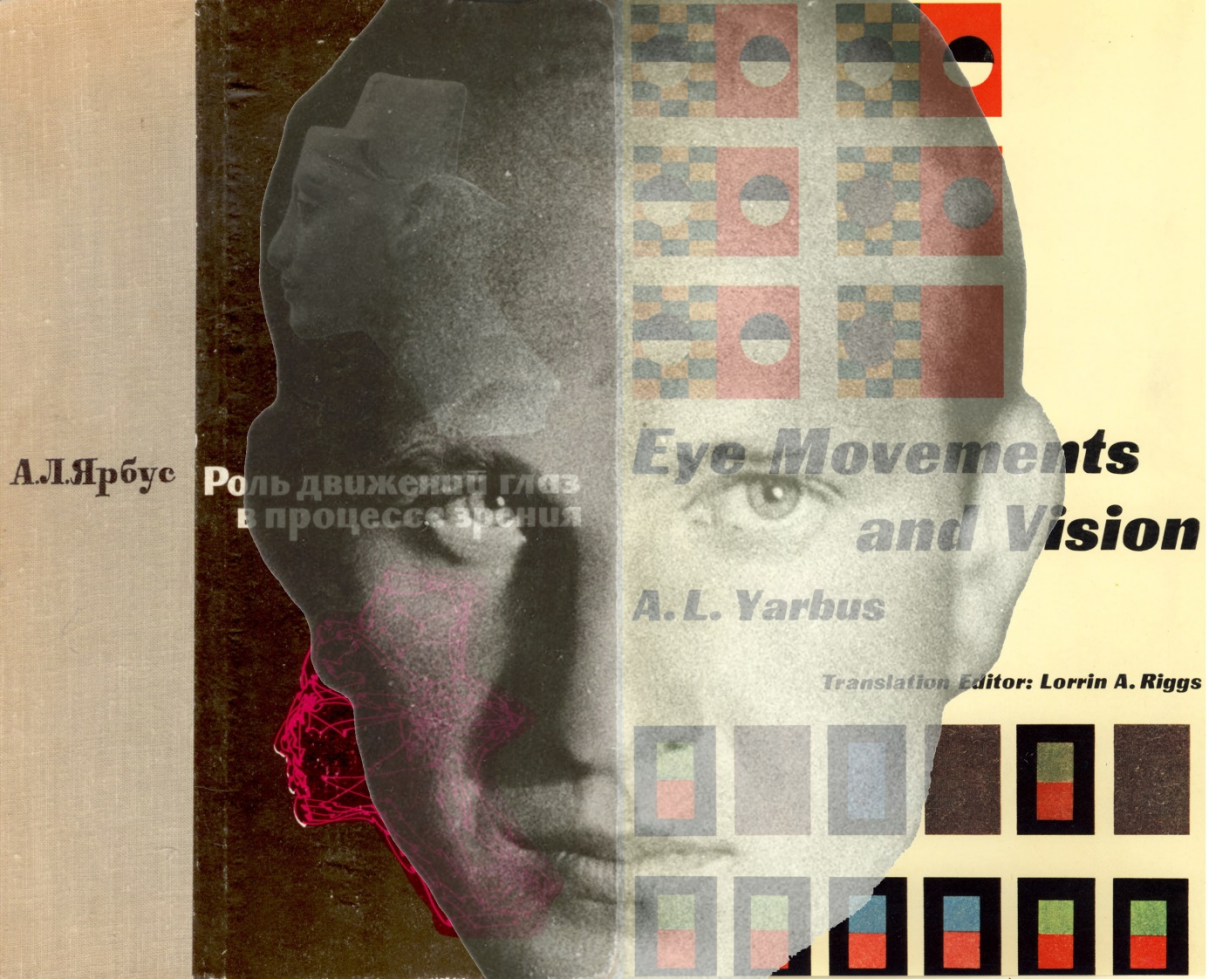
*Eye movements and Yarbus*. The covers of the Russian (31) and English (32) editions of Yarbus’s book are shown together with his portrait as a young man. (The cover of the Russian book and the portrait of Yarbus were kindly supplied by Galina Rozhkova.)

Buswell’s approach to eye movements found an echo in the work of Alfred Lukiyanovich Yarbus (Figure 12) three decades later. Yarbus commenced his research on visual process in the early 1950s (see 19; 22). His work was known to some Western scientists, in part because many of the articles were published in *Biofizik*a, a Russian journal whose first issue appeared in 1955, and which maintained an English-language translated version as *Biophysics*. Wider recognition was to follow the translation of his book *Eye Movements and Vision* into English (32); it opened the eyes of many researchers to the originality of his methods and to the implications of his experiments.

Yarbus also recorded eye movements over simple geometrical shapes like rectangles, triangles and ellipses (see 27; 29). These are rather like those examined by Stratton and Yarbus reached a similar conclusion: “Some readers may think that during the perception of stationary objects the human eyes are able to perform smooth pursuing movements in addition to saccades. Where stationary objects are concerned, this view is incorrect. It is due to the fact that the small saccades of the eyes are performed involuntarily and we are not aware of them” (32, p. 103). Like Judd, Yarbus examined eye movements and optical illusions. He was aware that “several authors have tried to explain the appearance of well- known optical illusions by movements of the eyes” but he did not say who the authors were. His first experiments involved presenting optically stabilised illusion figures (with the suction cap) and: “It was found that all the illusions persisted; consequently, they cannot be attributed to eye movements”. This was further supported by illuminating illusion figures with very brief flashes of light; not only were the illusions still present but they could still be seen in the afterimages from the bright light flashes. His final sober assessment of illusions applies as much today as it did when it was written: “The origin of many illusions is not yet known, however; the explanations advanced to date cannot be regarded as convincing. Different illusions influence eye movements to different degrees and in different ways. At the same time, some optical illusions have no influence whatever on eye movements” (32, p. 206; see also 27; 29).

Yarbus mentioned Buswell (6) in the context of reading, but did not cite his book in the more relevant area of eye movement patterns when looking at pictures (5). In fact, Yarbus did not read English and his access to foreign literature was very restricted ( 19; 33). Thus, the works in English and German that he cites must have been accessed through secondary sources or from his personal contacts. Within his book, Yarbus conducted an experiment where a single observer was shown the same painting seven times, but with a different question asked before each viewing. This elegant experiment confirmed Buswell’s earlier observation that the instructions given to an observer can radically change the places that the observer fixates. Yarbus’s demonstration has become a classic in eye movement research and is frequently cited as an unequivocal demonstration that high-level factors can over-shadow any low-level, stimulus-driven guidance of attention (see 8; 22).

**Figure 13.**
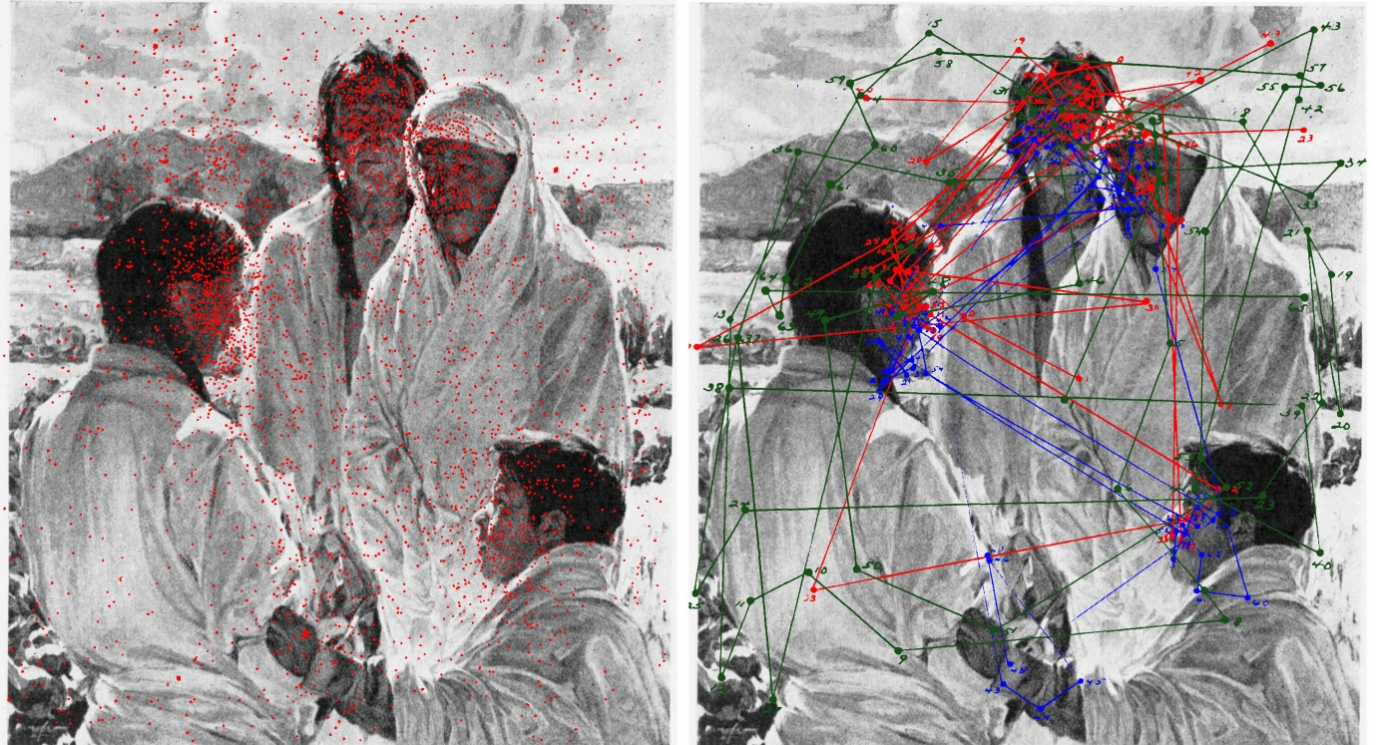
*Fixations and faces*. Left, locations of eye fixations (in red) for 76 observers over *The solemn pledge – Taos Indians* by Walter Ufer. Right, eye movement patterns of three observers (in red, green and blue) over the picture. (Adapted from Picture 12 and Plates IX, XXXV, XXXVI and XXXVII in Buswell, 5.)

Buswell (5) did include pictures of people in his set of stimuli although they were not full frontal face views as in Yarbus’s *Girl from the Volga*. When human figures were in the pictures viewed then fixations tended to concentrate on them. When the features of faces were present in the picture then they received more fixations than other regions. For example, during observation of a picture which included four figures he remarked “The outstanding characteristic of the pattern of perception at looking at this picture is the manner in which the fixations are concentrated over the four faces, a degree of concentration which leaves no doubt as to which are the principal centers of interest” (1935, p. 20). This can be seen in Figure 13.

**Figure 14.**
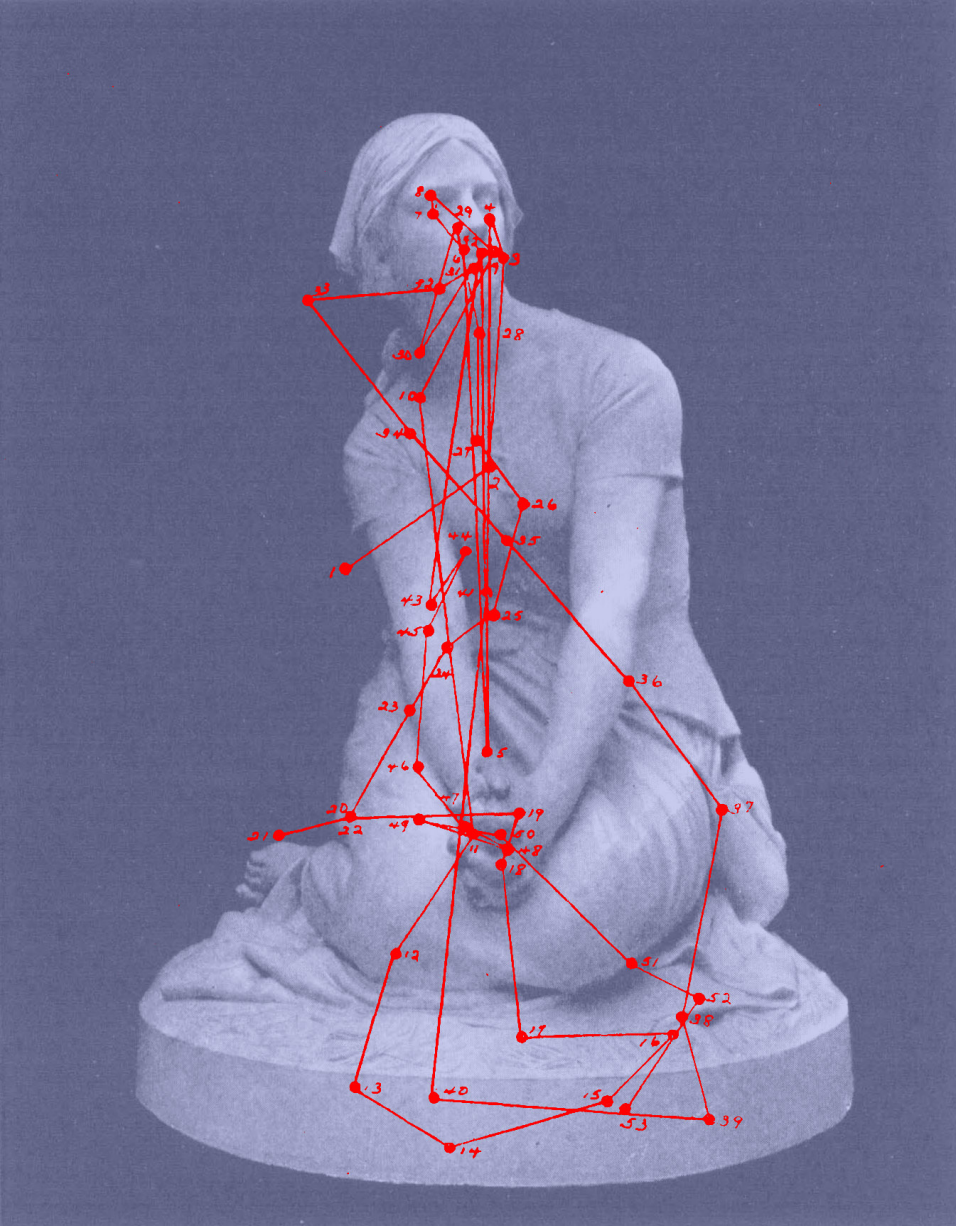
*Facial fixation*. Eye movements of one observer viewing a photograph of *Statue of Joan of Arc* by Henri-Michel-Antoine Chapu. (Adapted from Picture 31 and Plate XXXIV from Buswell, 5.)

In addition to these density plots, Buswell did examine the scan paths of individual observers and these provide clues to the scanning patterns found by Yarbus. With the figure mentioned above, as well as with observation of a photograph of a statue of Joan of Arc (Figure 14), there was evidence of fixations concentrated around the eyes and mouth. This picture was selected because of the absence of a patterned background. Although the scanning movements of only one observer were given Buswell stated that the eyes and hands were the centres of interest for most observers.

One of the enduring features of Yarbus’s investigations is the pattern of eye movements when viewing faces (or, more accurately, pictures of faces). His record of eye movements over the magazine picture of *Girl from the Volga* has been widely reproduced (see 15), and it is also shown (in red) superimposed on the face of Buswell in Figure 1. Eye tracks over a picture of the head of Queen Nefertiti was the basis for the cover illustration of the Russian edition of the book (see Figure 12). Yarbus wrote: “When looking at a human face, an observer usually pays most attention to the eyes, the lips, and the nose. The other parts of the face are given much more cursory consideration” (1967, p. 191). Because the eyes and lips are the most mobile and expressive features of a face, Yarbus considered that “it is absolutely natural and understandable that the eyes and lips attract the attention more than any other part of the human face” (1967, p. 191). He was surprised to find similar concentrations of fixations on a picture of a lion’s head and a sculpture of a gorilla.

**Figure 15.**
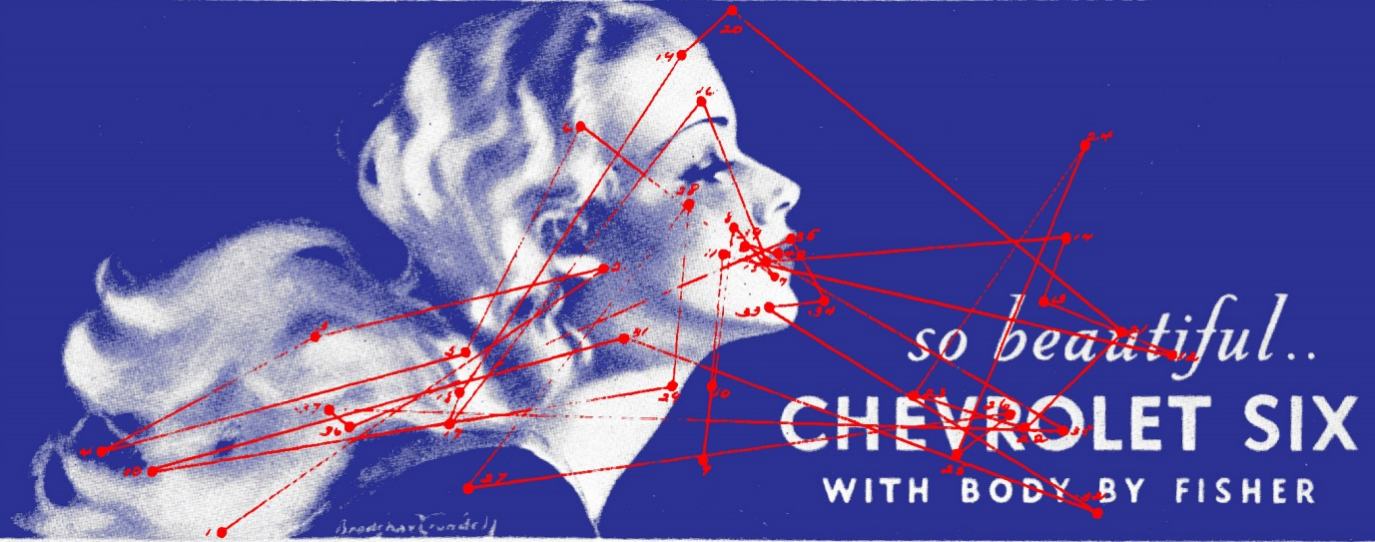
*Poster girl*. The sequence of saccades and the locations of fixations in observer 27 when viewing an advertising poster. (Adapted from Picture 48 and Plate LXVII in Buswell, 5.)

Buswell also used a profile face not unlike the picture of Nefertiti examined by Yarbus, but the additional text and extending flowing waves of hair resulted in the scan paths being more extended (Figure 15). Relatively few fixations were on the eye with most being around the lips. Buswell described it in the following way: “As would be expected, the picture of the girl was the principal center of interest. Not until the twelfth fixation was any attention given to the car which is advertised and then for a brief succession of three fixations. The eye returns again to the printing for Fixation 21, but swings back to the girl’s face and hair on Fixation 27” ( 5, p. 141). Buswell also appreciated the benefits that studies of eye movements could provide for advertisers and industry.

Face perception has become a major topic in vision studies (see 7). Faces not only fascinate us but they also provide vital clues to our social interactions. The scientific study of faces was revolutionised by photography in the mid-nineteenth century and facial expressions were photographed before aspects of face recognition were investigated (28). It has been further invigorated by investigations of eye movements like those of Buswell and Yarbus.

## Conclusion

Recording eye movements when looking at complex visual stimuli like pictures was a technical tour de force in 1935. The novelty of the investigations was appreciated by Buswell but the reader gets the impression from the text that there were no relevant studies before this. It is surprising that he did not cite any earlier studies, not even those of his mentor, Judd. The only references in the text were for the art historians whose quotations were given in his Introduction and for an art test. A similar strategy was adopted for his book on reading ( 6), an area in which there was a wealth of previous research (24).

Buswell carried out relatively little statistical analysis on the vast amount of data he collected. He let the density plots and eye tracking graphs talk for themselves. This probably reflects the general outcome of the investigations that individual differences swamp any small effects that might have been expected from the experimental manipulations he made. He certainly remained sanguine regarding the statements made by art historians and critics about eye movements and art. The same could be said about responses to Buswell’s book in art publications. In *The Burlington Magazine* the psychologist Robert Thouless (25) wrote “Our admiration for the ingenuity of Professor Buswell’s apparatus and the heroic laboriousness of his experiments and calculations may be a little tempered by our disappointment at noticing that nothing of the slightest importance to the sciences of æsthetics or psychology seems to result from this research” (p. 58). This says more about Thouless’s foresight than about Buswell’s book. Thouless did, however, make grudging acknowledgement of Buswell’s labours: “It is always better to observe accurately than to speculate, and anyone who wishes to defend or attack such theories in the future will find a solid foundation for his arguments in Professor Buswell’s work” (p. 58).

While the patterns of eye movements presented by Buswell are persuasive, providing some explicit rationale for selecting particular individual records for detailed consideration would have been welcome. For example, the dramatic effects of instructions on the patterns of eye movements (shown in Figure 9) are based on the data from a single individual. All that Buswell states is: “In selecting individual records for presentation the writer has tried to select those which are most typical of the main group of subjects, or which illustrate certain features to which particular attention is to be called” (p. 46).

Nonetheless, Buswell’s impressive monograph illustrates the rapid changes that were taking place in eye movement research in the first half of the twentieth century. Understanding of saccadic eye movements was rapidly increasing, as was the technology with which they could be measured; with these changes came both new questions about vision and the ability to address them in increasingly realistic viewing conditions and with increasing flexibility and precision. Buswell’s book discussed a wide range of issues regarding the relationship between eye movements and visual experience and these questions have been reflected in much of the eye movement research that has followed.

## Ethics and Conflict of Interest

The author declares that the contents of the article are in agreement with the ethics described in http://biblio.unibe.ch/portale/elibrary/BOP/jemr/ethics.html and that there is no conflict of interest regarding the publication of this paper.

With the exception of Figure 2 the illustrations were made by the author.
